# Non-linear Relationship Between Plasma Amyloid-β 40 Level and Cognitive Decline in a Cognitively Normal Population

**DOI:** 10.3389/fnagi.2020.557005

**Published:** 2020-09-11

**Authors:** Fan Gao, Suhang Shang, Chen Chen, Liangjun Dang, Ling Gao, Shan Wei, Jin Wang, Kang Huo, Meiying Deng, Jingyi Wang, Qiumin Qu

**Affiliations:** ^1^Clinical Research Center, The First Affiliated Hospital of Xi’an Jiaotong University, Xi’an, China; ^2^Department of Neurology, The First Affiliated Hospital of Xi’an Jiaotong University, Xi’an, China; ^3^Department of Neurology, Huxian Hospital of Traditional Chinese Medicine, Xi’an, China

**Keywords:** Alzheimer’s disease, plasma amyloid-β, cognitive decline, cognitively normal population, age

## Abstract

**Objectives:**

Recent studies regarding the relationships between plasma amyloid-β (Aβ) levels and cognitive performance had inconsistent results. In this study, we aimed to characterize the relationship between cognitive decline and plasma Aβ levels in a large-sample cognitively normal population.

**Methods:**

This population-based, prospective cohort study included 1,240 participants with normal cognition. The Mini-Mental State Examination (MMSE) was used to assess cognitive function at baseline and 2 years later. Restricted cubic splines, multivariate logistic regression, and multivariate linear regression models were used to evaluate the type of relationship between cognitive decline during the 2-year follow-up period and plasma Aβ levels (Aβ_40_, Aβ_42_, and Aβ_42__/__40_).

**Results:**

Participants with moderate Aβ_40_ levels had the highest risk of cognitive decline during a 2-year follow-up relative to individuals with low Aβ_40_ [odds ratio (OR): 0.60, 95% confidence interval (CI): 0.45–0.81, *p* < 0.001] or high Aβ_40_ (OR: 0.65, 95% CI: 0.49–0.87, *p* = 0.004) levels. The association between Aβ_40_ and cognitive decline did not depend on sex, education level, or APOE ε4 status. There was an interaction found between age (≤ 65 and > 65 years) and Aβ_40_ (*p* for interaction = 0.021). In individuals older than 65 years, there was a positive linear relationship between plasma Aβ_40_ and cognitive decline (OR: 1.02, 95% CI: 1.00–1.04, *p* = 0.027). For participants ≤ 65 years old, the lower Aβ_40_ and higher Aβ_40_ groups had a lower risk of cognitive decline than the medium Aβ_40_ group (OR: 0.69, 95% CI: 0.50–0.94, *p* = 0.02; OR: 0.63, 95% CI: 0.45–0.86, *p* = 0.004). None of relationship between plasma Aβ_42_, Aβ_42__/__40_ and cognitive decline was found during a 2-year follow-up.

**Conclusion:**

The relationship between plasma Aβ_40_ and cognitive decline was not linear, but an inverted-U shape in a cognitively normal population. The underlying mechanism requires further investigation.

## Introduction

Amyloid-β (Aβ) pathology has been confirmed as a pathological change in the early phase of Alzheimer ‘s disease (AD) (Sperling et al., 2011; Yaffe, 2011; Dubois et al., 2016; Jack et al., 2018; Nakamura et al., 2018; Jansen et al., 2018). Abnormal Aβ deposition in the brain increases the risk of cognitive decline and the development of dementia (Villemagne et al., 2011; van Harten et al., 2013; Jaunmuktane et al., 2015; Toledo et al., 2015; Donohue et al., 2017; Roberts et al., 2018; Greenberg et al., 2020). Accelerating our understanding of the relationship between amyloid pathology and cognitive functioning can contribute to the efficient screening of preclinical AD at an early stage, and considerably delay the progression of the disease (Jansen et al., 2018).

Amyloid pathology, visualized on positron emission tomography (PET) scans or measured in cerebrospinal fluid (CSF), has been extensively studied and has proven clinical applications for AD (Sperling et al., 2013; Vos et al., 2013; Toledo et al., 2015; Dubois et al., 2016; Olsson et al., 2016; Donohue et al., 2017). However, because these techniques are invasive or expensive, more economical and easily-acquired plasma Aβ measurements are needed. Recent studies have revealed that plasma Aβ are related to brain Aβ burden and incident dementia (Graff-Radford et al., 2007; Yaffe, 2011; Gabelle et al., 2013; Hanon et al., 2018; Hilal et al., 2018; Nakamura et al., 2018; Verberk et al., 2018; Chen et al., 2019; de Wolf et al., 2020), however, the relationships between plasma Aβ levels and cognitive performance were inconsistent. Previous studies reported that lower plasma Aβ_42__/__40_ or Aβ_42_ increased the risk of cognitive decline and the progression of dementia (Yaffe, 2011; Gabelle et al., 2013; Hilal et al., 2018; Verberk et al., 2018; de Wolf et al., 2020). Other studies have suggested that a positive relationship between plasma Aβ_42_, Aβ_42__/__40_ and cognitive impairment (Hanon et al., 2018; Wang et al., 2018; Chen et al., 2019).

In order to rapidly identify patients with cognitive decline earlier, and therefore delay the occurrence of AD, the present study aimed to determine the change in cognitive status, its relationship with plasma Aβ, and the type of association, during a 2-year follow-up in a cognitively normal population. This study also investigated whether this association was altered by sex, age, education level, or Apolipoprotein E (APOE) genotype, and estimated the relationship between plasma Aβ and cognitive disorder and cardio-cerebrovascular diseases during a 2-year follow-up.

## Materials and Methods

### Study Population

Participants were enrolled from a village in the suburbs of Xi’an city, located in northwestern China, between October, 2014 and November, 2015. This study was a community population cohort study in which each interviewee received a face-to-face questionnaire. Participants were asked about detailed history by neurologists, and received a nervous system examination, a systemic physical examination, blood biochemical examination aimed at determining whether there were any related neurological diseases. All patients with cerebral disease had received the CT or MRI scans. For patients who had a diagnosis of mild cognitive impairment (MCI) or dementia due to AD, we provided free MRI scan to confirm the diagnosis of AD in a local designated hospital. Inclusion criteria was that participants were (1) older than 40 years and (2) a current resident of the village and had lived there for more than 3 years. Exclusion criteria was that (1) the Mini-Mental State Examination (MMSE) was not completed; (2) there was evidence of MCI or dementia, or (3) other neurological conditions that may influence cognitive function (such as epilepsia, central nervous system infections, essential tremor, Parkinsonism, anxiety, depression, thyroid hypofunction, intracranial trauma, or surgery); (4) plasma Aβ was not measured; and (5) other baseline information was missing. This study was approved by the First Affiliated Hospital of Xi’an Jiaotong University. Written informed consent was obtained from all participants.

### Cognitive Evaluation

The MMSE was conducted as global cognitive screening at enrollment. The MMSE, other neurological, physical and imaging examinations were assessed at baseline and repeated 2 years later. Cutoff values were ≤ 17 for subjects with illiteracy, ≤20 for primary school educated subjects, and ≤ 24 for those educated at the junior high school level or above (Bohm et al., 2015). After initial screening, study participants with MMSE score ≤ these cutoff values underwent the 2nd phase cognitive examinations, including the Fuld Object Memory Evaluation test, Rapid Verbal Retrieval test, Trail-Making test, Digit Span test, and Block Design test. Finally, combining all the available imaging and clinical information, the diagnosis and subtype of dementia and MCI were determined according to international diagnostic criteria for dementia (American Psychiatric Association [APA], 2000) and Petersen MCI criteria (Petersen, 2011) by a panel of senior clinicians. The diagnosis of AD was based on NINCDS-ADRDA criteria (McKhann et al., 2011).

### Laboratory Measurement

All plasma samples were acquired between 7 and 11 a.m. Plasma Aβ_40_ and Aβ_42_ tests were measured using enzyme-linked immunosorbent assay kits (ELISA, Yuanye Co. Shanghai, China) (Wei et al., 2017; Jiang et al., 2018). Concentrations of Aβ_40_ and Aβ_42_ were conducted in duplicate and determined from standard curves using a Rayto RT-6000 analyzer (Rayto Co., Shenzhen, China) at OD 450 nm. Apolipoprotein E (APOE) was genotyped by polymerase chain reaction (PCR), followed by sequencing, as previously described (Wenham et al., 1991).

### Outcome Definition

The primary outcome was cognitive decline, which was defined as the difference of MMSE between the first visit and the second visit > 0. Cognitive disorder was defined as MMSE score ≤ 17 for illiterate, ≤ 20 for primary school educated, and ≤ 24 for junior high school educated or above (Katzman et al., 1988). New cardiovascular and cerebrovascular diseases were defined as incident cardiovascular and cerebrovascular diseases during the 2-year follow-up.

### Other Covariates

Lack of physical exercise was defined as exercising fewer than three times a week, fewer than 30 min a time. Smoking was defined as a current smoker. Diabetes mellitus was defined as a self-reported medical diagnosis, diabetic medication use, a fasting glucose of ≥ 7.0 mmol/l, a random plasma glucose concentration ≥ 11.1 mmol/l, or glycated hemoglobin ≥ 6.5%. Hypertension was defined as a self-reported medical diagnosis, antihypertensive medication use, a systolic blood pressure of ≥ 140 mmHg, or a diastolic blood pressure of ≥ 90 mmHg.

### Statistical Analysis

Continuous variables are presented as mean ± SD, and categorical variables are presented as proportions. We determined the association between baseline information and cognitive decline using Pearson’s χ^2^ or Fisher’s exact test for categorical variables, and Student’s *t*-test or Mann–Whitney *U*-test for continuous variables. Differences of basic characteristics between different Aβ groups were compared using Pearson’s χ^2^ or Fisher’s exact test for categorical variables, and ANOVA test or Kruskal-Wallis H test for continuous variables.

Restricted cubic splines with a logistic regression model were applied to test the relationship between cognitive decline and Aβ levels, and to evaluate the type of correlation of cognitive decline with Aβ levels. Four knots defined at the 5th, 35th, 65th, and 95th percentiles of Aβ levels were prespecified (Harrell, 2001). The reference point was the mean value of Aβ levels. Aβ categories were based on the type of relationship between cognitive decline, and Aβ levels suggested in restricted cubic splines. A series of pre-planned categories of Aβ levels were tested, defined by tertiles and interquartiles. The differences in MMSE scores between baseline and 2 years later were compared with the paired *t*-test.

To assess the association between Aβ levels (categorical and continuous) and various outcomes, unadjusted, age- and sex-adjusted, and multivariable-adjusted logistic regression models were used. A multivariable linear regression model was used to evaluate the relationship between Aβ and 2-year MMSE score change. In multivariable models, adjustments were made for age, sex, diabetes, hypertension, smoking, lack of physical activity, APOE ε4 status, and education. In subgroup analyses, we examined the relationship between Aβ_40_ and cognitive decline by sex, age (≤ 65 and > 65 years), education (≥ high school and < high school), and APOE ε4 status in multivariable-adjusted logistic regression models. SPSS ver. 24.0 and R ver. 3.5.3 were used for statistical analysis. A *p* < 0.05 was considered significant.

## Results

### Characteristics of the Study Population at Baseline

**FIGURE 1:**
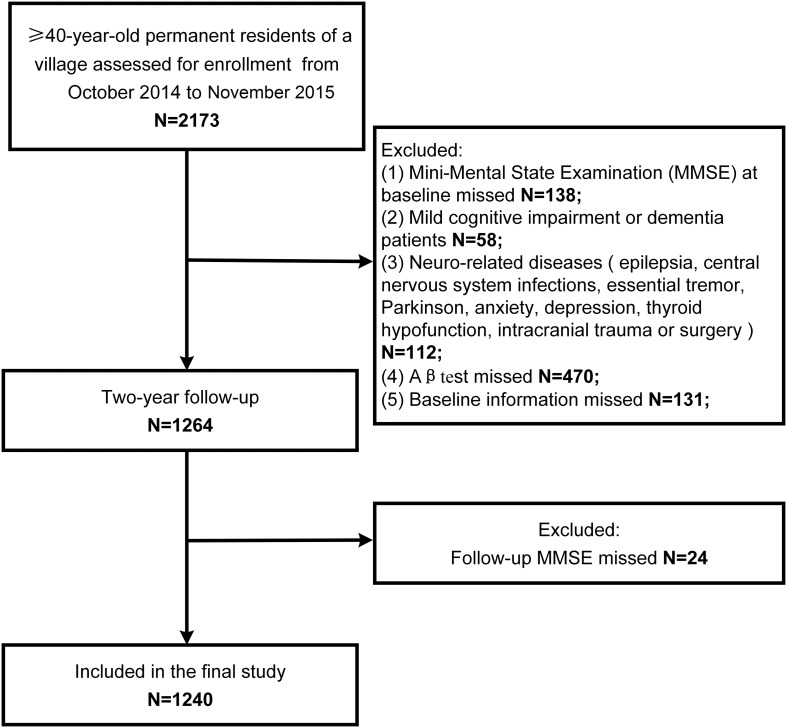
Study population screening process.

The study population screening process is shown in Figure 1. There were 2,173 individuals examined from October, 2014 to November, 2015. A total of 138 participants did not complete the MMSE, 58 had MCI or dementia, 112 had neurologically-related diseases, 470 did not undergo plasma Aβ tests, 131 had missing baseline information, and 24 did not complete the follow-up MMSE. Finally, 1,240 participants were included in our study.

Baseline characteristics of the study population are listed in Table 1. Among 1240 participants, 456 experienced cognitive decline. The mean age of the study participants was 55.2 ± 9.7 years and 469 (37.8%) were men. In the group without cognitive decline, the percentage of higher education level was higher (63.3%) than that in the group with cognitive decline (55.3%, *p* = 0.005). MMSE score was higher (27 ± 3.2) in the group with cognitive decline than that in the population without cognitive decline(25.5 ± 3.7, *p* < 0.001. There were no other significant differences between two groups.

**TABLE 1:** Baseline characteristics according to cognitive decline.

**Variable**	**Cognitive decline *n* = 456**	**Without cognitive decline *n* = 784**	***p***
Age (y)	56 ± 10	54.8 ± 9.4	0.048
Male, n(%)	186 (40.8)	283 (36.1)	0.100
Education ≥ high school, n(%)	252 (55.3)	496 (63.3)	0.005
Diabetes, n(%)	39 (8.6)	62 (7.9)	0.689
Hypertension, n(%)	134 (29.4)	217 (27.7)	0.520
Smoking, n(%)	136 (29.8)	197 (25.1)	0.072
Alcohol drinker, n(%)	63 (13.8)	103 (13.1)	0.735
Lack of exercise, n(%)	92 (20.2)	124 (15.8)	0.051
SBP (mmHg)	133.6 ± 19.2	132.1 ± 18.2	0.112
Pulse (/min)	75.2 ± 8.4	75.7 ± 9.5	0.728
BMI (kg/m^2^)	25.2 ± 3.2	25.4 ± 3.2	0.197
TC (mmol/L)	5 ± 1	5 ± 1	0.892
TG (mmol/L)	1.6 ± 0.9	1.6 ± 0.9	0.588
HDL (mmol/L)	1.4 ± 0.3	1.4 ± 0.3	0.19
LDL (mmol/L)	3.3 ± 0.9	3.3 ± 0.9	0.829
APOE ε4 positive, n(%)	64 (14)	102 (13)	0.609
Aβ_40_ (pg/mL)	50 ± 14.2	49.7 ± 15.1	0.645
Aβ_42_ (pg/mL)	41.1 ± 6.8	41 ± 6.5	0.963
Aβ_42__/__40_ (pg/mL)	9.1 ± 36.7	9.9 ± 38.2	0.915
MMSE at baseline	27 ± 3.2	25.5 ± 3.7	<0.001

*SBP, systolic blood pressure; BMI, body mass index; TC, total cholesterol; TG, triglycerides; HDL, high-density lipoprotein cholesterol; LDL, low-density lipoprotein cholesterol; APOE, apolipoprotein E; A*β, *Amyloid-*β*; MMSE, Mini-Mental State Examination.*

### Association Between Continuous Aβ_40_, Aβ_42_, Aβ_42__/__40_ Levels and Cognitive Decline

The associations between continuous Aβ_40_, Aβ_42_, Aβ_42__/__40_ levels and cognitive decline were listed in Table 2. After multivariable adjustment, none of three biomarkers reached statistical significance (all *p* > 0.05).

**TABLE 2:** Association of plasma levels of amyloid-β 40, amyloid-β 42, and the amyloid-β 42/amyloid-β 40 ratio with cognitive decline.

**Biomarker**	**Association with cognitive decline**					

	**Unadjusted**		**Age-and sex-adjusted**		**Fully adjusted^a^**	
	**OR (95%CI)**	***p***	**OR (95%CI)**	***p***	**OR (95%CI)**	***p***
Aβ_40_	1.00	0.752	1.00	0.786	1.00	0.731
(per pg/mL increase)	(0.99–1.01)		(0.99–1.01)		(0.99–1.01)	
Aβ_42_	1.00	0.738	1.00	0.827	1.00	0.770
(per pg/mL increase)	(0.99–1.02)		(0.99–1.02)		(0.99–1.02)	
Aβ_42__/__40_	1.00	0.725	1.00	0.698	1.00	0.579
	(1.00–1.00)		(1.00–1.00)		(1.00–1.00)	

*^*a*^Fully adjusted for age, sex, diabetes, hypertension, smoking, lack of physical exercise, APOE*ε*4, education* ≥ *high school. OR, odds ratio; CI, confidence interval.*

**FIGURE 2:**
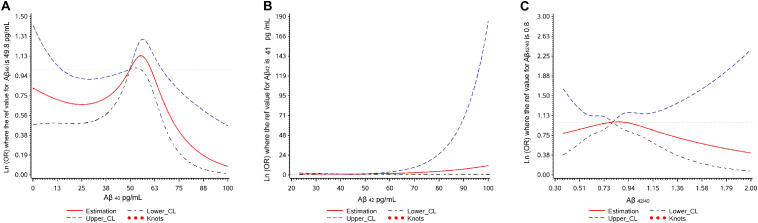
Plot of dose-response relationship between plasma Aβ levels and cognitive decline during two-year follow-up.

The associations between Aβ levels and cognitive decline using restricted cubic splines are shown in Figure 2. Aβ_40_ showed a non-linear relationship (*p* overall = 0.040, *p* non-linear = 0.017) with cognitive decline. Aβ_42_ and Aβ_42__/__40_ didn’t show any relationship with cognitive decline (*p* overall > 0.05; *p* non-linear > 0.05).

### Non-linear Relationship Between Plasma Aβ_40_ Level and Cognitive Decline

Because of the non-linear relationship between plasma Aβ_40_ and cognitive decline, and the inverted U shape found in the restricted cubic spline, we transformed continuous Aβ_40_ into two kinds of categorical variables: three tertiles defined by the 33rd percentiles and 66th percentiles, and 3-class classified variables defined by the 25th and 75th percentiles. In multivariate logistic regression analyses evaluating the relationship between cognitive decline and categorical Aβ_40_, 3-classified Aβ_40_ had a better goodness of fit (χ^2^ = 32.20, *p* < 0.001) than Aβ_40_ defined by the 33rd and 66th percentiles (χ^2^ = 15.44, *p* = 0.117). Therefore, 3-classified Aβ_40_, defined by the 25th and 75th percentiles, was used in the present study.

**FIGURE 3:**
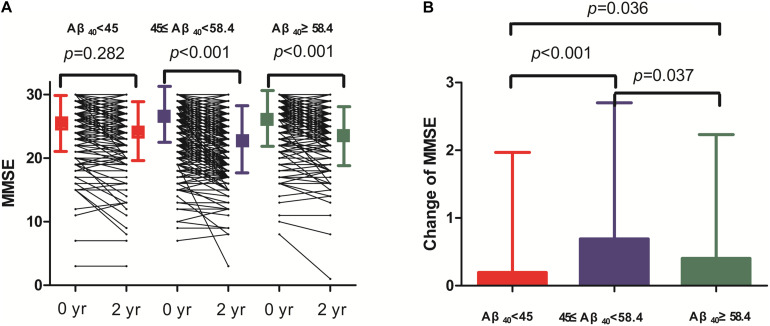
The changes of cognitive function during 2 year follow-up.

Two-year changes in MMSE score in the three Aβ_40_ groups are shown in Figure 3. Paired comparisons of MMSE scores between baseline and 2 years later in the medium and high Aβ_40_ groups were significant (*p* < 0.001, Figure 3A). When plasma was 45 pg/mL ≤ Aβ_40_ < 58.4 pg/mL, the change in MMSE scores between the 2 visits was more evident than in the other 2 groups (Figure 3B). The changes in MMSE scores in the low, medium, and high Aβ_40_ groups were 0.09 ± 1.53, 0.65 ± 1.89, and 0.39 ± 1.78 pg/mL respectively. Baseline characteristics of the study population, stratified by low (< 45 pg/mL), medium (45 pg/mL ≤ Aβ_40_ < 58.4 pg/mL), and high Aβ_40_ (≥58.4 pg/mL) are listed in Supplementary Table S1.

The relationship between plasma Aβ_40_ and various outcomes during follow-up using multivariable logistic regression models are listed in Table 3. In unadjusted analyses, compared with participants with Aβ_40_ between 45 and 58.4 pg/mL, participants in the low Aβ_40_ group and high Aβ_40_ group had lower risks of cognitive decline (OR: 0.61, 95% CI: 0.46–0.82, *p* < 0.001; OR: 0.67, 95% CI: 0.50–0.89, *p* = 0.006). After adjustments for age and sex, the results were the same as before. In the fully adjusted model, participants in the low Aβ_40_ (< 45 pg/mL) and high Aβ_40_ groups (≥58.4 pg/mL) still had a lower risk of cognitive decline, compared with the medium Aβ_40_ group (OR: 0.60, 95% CI: 0.45–0.81, *p* < 0.001; OR: 0.65, 95% CI: 0.49–0.87, *p* = 0.004). In addition, participants in the low Aβ40 group had a lower risk of a new cognitive disorder (OR: 0.29, 95% CI: 0.12–0.70, *p* = 0.006) than participants in the medium Aβ_40_ group, while participants with high Aβ_40_ levels did not show this association. There were no significant associations found in other outcomes. The relationships of categorical Aβ_40_ defined by the 33th percentiles and 66th percentiles, and cognitive decline are listed in Supplementary Table S2.

**TABLE 3:** Results of multivariable logistic regression models examining the relationship between plasma Aβ_40_ and various outcomes during follow-up.

**Outcomes**	**No. of events (%)**	**Unadjusted**			**Age- and sex-adjusted**			**Fully adjusted^a^**		
		**OR**	**95%CI**	***p***	**OR**	**95%CI**	***p***	**OR**	**95%CI**	***p***
**Cognitive decline**										
< 45 pg/mL	95 (30.65)	0.61	0.46–0.82	< 0.001	0.61	0.46–0.82	0.001	0.60	0.45–0.81	< 0.001
45∼58.4 pg/mL	260 (41.94)	1.0 (ref)			1.0 (ref)			1.0 (ref)		
≥ 58.4 pg/mL	101 (32.58)	0.67	0.50–0.89	0.006	0.67	0.50–0.89	0.006	0.65	0.49–0.87	0.004
**New cognitive disorder**										
< 45 pg/mL	6 (1.94)	0.31	0.13–75	0.009	0.31	0.13–0.74	0.009	0.29	0.12–0.70	0.006
45∼58.4 pg/mL	37 (5.97)	1.0 (ref)			1.0 (ref)			1.0 (ref)		
≥ 58.4 pg/mL	14 (4.52)	0.75	0.40–1.40	0.361	0.72	0.38–1.36	0.305	0.70	0.37–1.32	0.270
**Cognitive disorder at baseline**										
< 45 pg/mL	34 (10.97)	1.41	0.89–2.22	0.147	1.43	0.90–2.28	0.131	1.41	0.88–2.26	0.151
45∼58.4 pg/mL	50 (8.06)	1.0 (ref)			1.0 (ref)			1.0 (ref)		
≥ 58.4 pg/mL	28 (9.03)	1.13	0.70–1.84	0.616	1.1	0.67–1.80	0.706	1.10	0.67–1.80	0.710
**New cardiovascular diseases**										
< 45 pg/mL	19 (6.13)	0.8	0.46–1.38	0.414	0.8	0.45–1.39	0.421	0.81	0.46–1.42	0.452
45∼58.4 pg/mL	47 (7.59)	1.0 (ref)			1.0 (ref)			1.0 (ref)		
≥ 58.4 pg/mL	16 (5.16)	0.66	0.37–1.19	0.167	0.63	0.35–1.14	0.124	0.64	0.35–1.16	0.138
**New cerebrovascular diseases**										
< 45 pg/mL	8 (2.58)	0.49	0.22–1.07	0.072	0.48	0.22–1.07	0.072	0.49	0.22–1.08	0.077
45∼58.4 pg/mL	32 (5.17)	1.0 (ref)			1.0 (ref)			1.0 (ref)		
≥ 58.4 pg/mL	14 (4.52)	0.87	0.46–1.65	0.665	0.84	0.44–1.60	0.588	0.86	0.44–1.66	0.644

*OR, odds ratio; CI, confidence interval; A*β, *Amyloid-*β. *^*a*^Fully adjusted for age, sex, diabetes, hypertension, smoking, lack of physical exercise, APOE*ε*4, education* ≥ *high school.*

**FIGURE 4:**
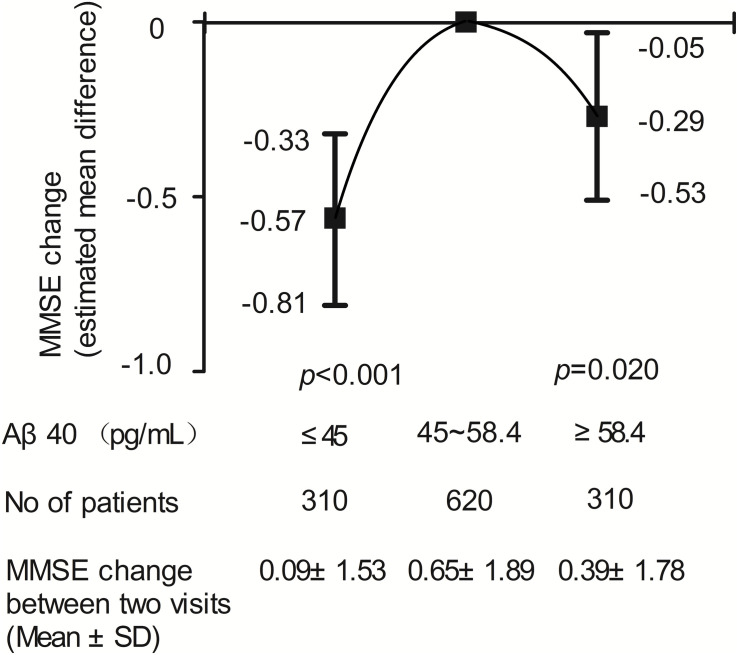
Results of multivariable linear regression analysis evaluating the relation between plasma Aβ_*40*_ and cognitive decline during two-year follow-up.

The relationship between plasma Aβ_40_ and 2-year change in MMSE score using a multivariate linear regression model is shown in Figure 4. An inverted U-shaped association between Aβ_40_ and MMSE score change was still apparent. Compared with participants with medium Aβ_40_, those with low Aβ_40_ and high Aβ_40_ had a lower risk of MMSE score change (β: −0.57, 95% CI: −0.81 to −0.33, *p* < 0.001; β: −0.29, 95% CI: −0.53 to −0.05, *p* = 0.020).

### Subgroup Analyses of the Relationship Between Plasma Aβ_40_ and Cognitive Decline

Subgroup analyses of the relationship between plasma Aβ_40_ and cognitive decline are listed in Table 4. The association between Aβ_40_ and cognitive decline did not depend on sex, education level, or APOE ε4 status. There was an interaction found between age (≤ 65 and > 65 years) and Aβ_40_ (*p* for interaction = 0.021). For participants ≤ 65 years old, the lower Aβ_40_ and higher Aβ_40_ groups had a lower risk of cognitive decline than the medium Aβ_40_ group (OR: 0.69, 95% CI: 0.50–0.94, *p* = 0.02; OR: 0.63, 95% CI: 0.45–0.86, *p* = 0.004). In participants aged > 65 years, lower Aβ40 levels correlated with cognitive decline (OR: 0.29, 95% CI: 0.14–0.63, *p* = 0.002) compared with the medium Aβ_40_ group. Continuous Aβ_40_ level was associated with the increased risk of cognitive decline (OR: 1.02, 95%CI: 1.00–1.04, *p* = 0.027).

**TABLE 4:** Subgroup analyses of relationship between plasma Aβ_40_ and cognitive decline.

**Subgroups**	**No. of events (%)**	**OR^a^**	**95%CI**	***p***	***p* interaction**
Sex					0.323
Male					
Continuous	186 (39.7)	1.01	0.99–1.02	0.266	
< 45 pg/mL	33 (29.5)	0.41	0.25–0.68	< 0.001	
45∼58.4 pg/mL	116 (47.9)	1.0 (ref)			
≥ 58.4 pg/mL	37 (32.2)	0.43	0.27–0.71	< 0.001	
Female					
Continuous	270 (35.0)	1.00	0.99–1.01	0.698	
< 45 pg/mL	62 (31.3)	0.74	0.52–1.07	0.115	
45∼58.4 pg/mL	144 (38.1)	1.0 (ref)			
≥ 58.4 pg/mL	64 (32.8)	0.8	0.55–1.15	0.219	
Age					0.021
Age ≤ 65					
Continuous	367 (35.6)	1.00	0.99–1.01	0.380	
< 45 pg/mL	82 (31.7)	0.69	0.50–0.94	0.020	
45∼58.4 pg/mL	208 (40.3)	1.0 (ref)			
≥ 58.4 pg/mL	77 (30.1)	0.63	0.45–0.86	0.004	
Age > 65					
Continuous	89 (45.6)	1.02	1.00–1.04	0.027	
< 45 pg/mL	13 (25.5)	0.29	0.14–0.63	0.002	
45∼58.4 pg/mL	52 (50)	1.0 (ref)			
≥ 58.4 pg/mL	24 (44.4)	0.72	0.36–1.44	0.358	
Education ≥ high school					0.461
Yes					
Continuous	252 (33.7)	1.00	0.99–1.02	0.520	
< 45 pg/mL	51 (27.4)	0.6	0.41–0.88	0.009	
45∼58.4 pg/mL	150 (39.0)	1.0 (ref)			
≥ 58.4 pg/mL	51 (28.8)	0.63	0.43–0.93	0.021	
No					
Continuous	204 (41.5)	1.00	0.99–1.01	0.859	
< 45 pg/mL	44 (35.5)	0.61	0.39–0.97	0.035	
45∼58.4 pg/mL	110 (46.8)	1.0 (ref)			
≥ 58.4 pg/mL	50 (37.6)	0.67	0.43–1.04	0.073	
APOE ε4 carrier					0.186
Yes					
Continuous	64 (38.6)	1.01	0.99–1.04	0.257	
< 45 pg/mL	9 (20.5)	0.26	0.11–0.63	0.003	
45∼58.4 pg/mL	40 (48.8)	1.0 (ref)			
≥58.4 pg/mL	15 (37.5)	0.65	0.29–1.43	0.284	
No					
Continuous	392 (36.5)	1.00	0.99–1.01	0.834	
<45 pg/mL	86 (32.3)	0.68	0.50–0.93	0.017	
45∼58.4 pg/mL	220 (40.9)	1.0 (ref)			
≥ 58.4 pg/mL	86 (31.9)	0.66	0.48–0.90	0.008	

*OR, odds ratio; CI, confidence interval; APOE, apolipoprotein E; A*β, *Amyloid-*β. *^a^Logistic regression models were used and adjusted for age, sex, diabetes, hypertension, smoking, lack of physical exercise, APOE*ε*4 and education* ≥ *high school.*

## Discussion

In this large, prospective, population-based cohort study, we found an inverted U-shaped relationship between plasma Aβ_40_ and cognitive decline during a 2-year follow-up in individuals with normal cognition. People with medium plasma Aβ_40_ concentrations (45–58.4 pg/mL) had the highest risk of cognitive decline than persons with plasma Aβ_40_ < 45 pg/mL and ≥ 58.4 pg/mL.

Previous studies have shown evidence of an association between plasma Aβ levels and the progression of AD, although most studies focused on plasma Aβ_42_ and plasma Aβ_42__/__40_ ratio (Schupf et al.; Graff-Radford et al., 2007; Yaffe, 2011; Gabelle et al., 2013; de Rojas et al., 2018; Hanon et al., 2018; Hilal et al., 2018; Verberk et al., 2018; Chen et al., 2019). Hanon et al. (2018) did not find a significant linear association between Aβ_40_ and MMSE score in their prospective cohort study, which included 1,040 participants. Several prospective cohort studies found that plasma Aβ_42_ or Aβ_42__/__40_ ratio was associated with an increased risk of developing AD, while no associations of this kind were found for plasma Aβ40 (Graff-Radford et al., 2007; Schupf et al., 2008; Yaffe, 2011; Gabelle et al., 2013; de Rojas et al., 2018; Hilal et al., 2018; Verberk et al., 2018). Chen et al. reported a linear relationship between plasma Aβ_42_ and MMSE score, regardless of plasma Aβ_40_ (Chen et al., 2019). This is the first study to report a non-linear relationship between plasma Aβ_40_ and cognitive decline. The reason may be attributed to different study population and different follow-up time. Most of previous studies observed marked cognitive decline (clinical progression to MCI) or the incidence of dementia for a long follow-up time in old and high-risk dementia populations (Yaffe, 2011; Gabelle et al., 2013; Verberk et al., 2018; de Wolf et al., 2020). In our study, we just observed 2-year MMSE change in a cognitively normal population with middle and old age. Two prior studies ever reported that higher plasma Aβ_40_ level was correlated with a decreased hippocampal volume (Kaffashian et al., 2015; Hanon et al., 2018). There was also evidence to reveal that Aβ levels were linked with brain atrophy only at an initial stage before cognitive impairment. The relationship between plasma Aβ levels and brain structural variation or cognitive outcomes during earlier stage of AD progression or normal cognitive aging may be different with the association at later stages (Hanon et al., 2018). Therefore, the association between plasma Aβ_40_ and cognitive decline may be earlier detected than the relationship between plasma Aβ_42_ and cognition progression due to a shorter observation period (2-year follow-up) and younger population in our study. It is why we only find the correlation of cognitive decline with plasma Aβ_40_, not plasma Aβ_42_.

The precise mechanisms underlying the relationship between plasma Aβ_40_ and cognitive decline remain unclear. There may be both of neurodegenerative and cerebrovascular pathologies promoting cognitive impairment in the study participants (Greenberg et al., 2020). Aβ_40_ was reported to be associated with the development of vascular dementia as well as AD (Hansson et al., 2012). Compared with moderate plasma Aβ_40_ group, higher plasma Aβ_40_ level may indicate more Aβ efflux from the brain to the peripheral blood and fewer deposition in brain. Therefore, the cognitive decline in the high plasma Aβ_40_ group may be slower. Besides, although the concentration of Aβ_40_ measured in peripheral blood was not high in the moderate Aβ_40_ group, the deposit of Aβ_40_ in the wall of vessels may be relatively high, which was involved with the development of vascular dementia. Compared with moderate Aβ_40_ group, low concentration of plasma Aβ_40_ may suggest increased degradation of Aβ_40_ by peripheral tissues, such as liver and kidney and decreased production of Aβ_40_ from brain and peripheral tissues (Kaffashian et al., 2015). It could reflect favorable condition of blood vessels, liver and kidney, which was significant to maintain normal cognitive function.

The characteristics of Aβ_40_ is different with Aβ_42__._ Aβ_40_ is more soluble than Aβ_42_, which can diffuse along perivascular drainage pathways to deposit in the walls of vessels, while Aβ_42_ tends to retained in the brain parenchyma and initiate insoluble plaque nucleation (Greenberg et al., 2020). In addition, Aβ_40_ is produced or degraded by peripheral tissues as well as Aβ_40_ produced and deleted in the brain (Kaffashian et al., 2015). Plasma Aβ_40_ may be not a direct surrogate marker for cerebral Aβ_40_, which can’t reflect Aβ_40_ and Aβ_42_ metabolism in the brain (Sonnen et al., 2008). Accordingly, the effect of Aβ_40_ on the physiologic process of dementia may be more complicated than Aβ_42__._ Because this study is a community-based cohort study, which failed to measure the liver and kidney function comprehensively, nor did it assess the deposition of Aβ in the brain parenchyma and vascular wall. The mechanisms of plasma Aβ_40_ affecting cognitive performance still needs further studies.

Of note, we found that the inverted U-shaped relationship between plasma Aβ_40_ and cognitive decline was more marked for young people (40–65 years old), while there seems to be a positive linear relationship between plasma Aβ_40_ and cognitive decline in individuals older than 65 years. This result agrees with other studies that suggested that amyloid-cognition association varied by age (Rowe et al., 2010; Rentz et al., 2011; Sperling et al., 2013; Petersen et al., 2016; Jansen et al., 2018). Jansen et al. (2018) found that the relationship between higher amyloid deposition in the brain and low memory scores was more evident in individuals older than 70 years old. Coincidentally, another recent study found no association between continuous PET-Aβ levels and memory scores in younger people (50–69 years old) (Mielke et al., 2016). In older population (> 65 years old), our results supported the previous studies (Hansson et al., 2012; Kaffashian et al., 2015; Hanon et al., 2018). For younger individuals (40–65 years old), our studies suggested that conventional evaluation methods (continuous or dichotomized Aβ_40_) were not suitable for assessing the relationship between plasma Aβ_40_ and cognitive decline.

There are still limitations that need to be considered. First, only the total MMSE score was evaluated for every participant in this study; sub-items of MMSE and other cognitive scores should be included to confirm cognitive decline. Second, ELISA is not the most sensitive technique for assessing plasma Aβ levels. Recent studies have employed single-molecule array or hybrid mass spectrometry techniques which may be more accurate approaches than ELISA (Nakamura et al., 2018; Vergallo et al., 2019). Nevertheless, ELISA is suitable for epidemiological studies with large sample size because of simple operation and cheapness. Third, the correlation of plasma Aβ_40_ with CSF Aβ_40_ and brain Aβ_40_, and the mechanism of transportation and clearance of Aβ_40_, were not studied. It would be useful to explore the dynamic transportation mechanism of Aβ_40_ between the brain and peripheral blood in future studies, in order to verify the present results.

## Conclusion

This population-based, prospective cohort study showed that the relationship between plasma Aβ_40_ and cognitive decline is not linear, but an inverted-U shape, in a cognitively normal population. Medium concentration of plasma Aβ_40_ is associated with a greater risk of cognitive decline, compared with low and high levels of plasma Aβ_40_, primarily in younger persons (≤65 years old). The underlying mechanism needs to be further elucidated.

## Data Availability Statement

The raw data supporting the conclusions of this article will be made available by the authors, without undue reservation, to any qualified researcher.

## Ethics Statement

The studies involving human participants were reviewed and approved by the ethics committee for medical research at the First Affiliated Hospital of Xi’an Jiaotong University. The patients/participants provided their written informed consent to participate in this study.

## Author Contributions

FG and QQ: conception and design of the study. SS, CC, LD, LG, SW, JW, and QQ acquisition of the data. FG, SS, and CC: analysis of data. FG, JW, KH, MD, and QQ interpretation of data. FG: wrote the first manuscript draft. SS, CC, LD, LG, SW, JW, KH, MD, JW, and QQ: revised the manuscript for intellectual content. All authors contributed to the article and approved the submitted version.

## Conflict of Interest

The authors declare that the research was conducted in the absence of any commercial or financial relationships that could be construed as a potential conflict of interest.
