# Supplementary material for: Non-linear Relationship Between Plasma Amyloid-β 40 Level and Cognitive Decline in a Cognitively Normal Population
Source: Front Aging Neurosci. 2020 Sep 11;12:557005. doi: 10.3389/fnagi.2020.557005 (PMC7516983; doi:10.3389/fnagi.2020.557005)
Supplement: Supplementary file 1 [file Table_1.docx]

**Table S1.** Baseline characteristics according to the category of plasma Aβ_40_.

| Variable | Low <45 pg/mL | Medium 45~58.4 pg/mL | High ≥58.4 pg/mL | *p* value |
| --- | --- | --- | --- | --- |
|  | n=310 | n=620 | n=310 |  |
| Age (y) | 55.1±9.5 | 55.2±9.6 | 55.6±9.9 | 0.747 |
| Male, n(%) | 112 (36.1) | 242(39.0) | 115 (37.1) | 0.659 |
| Education ≥high school, n(%) | 186(60) | 385(62.1) | 177 (57.1) | 0.337 |
| Diabetes, n(%) | 16(5.2) | 53(8.6) | 32(10.3) | 0.055 |
| Hypertension, n(%) | 89(28.7) | 176(28.4) | 86(27.7) | 0.963 |
| Smoking, n(%) | 89(28.7) | 163(26.3) | 81(26.1) | 0.695 |
| Alcohol drinker, n(%) | 38(12.3) | 88(14.2) | 40(12.9) | 0.687 |
| Lack of exercise, n(%) | 49(15.8) | 108(17.4) | 59(19.0) | 0.571 |
| SBP (mmHg) | 131.7±17.9 | 132.5±19.0 | 133.9±18.6 | 0.315 |
| Pulse (/min) | 75.1±8.4 | 75.9±9.4 | 75.3±9.2 | 0.424 |
| BMI (kg/m^2^) | 25.4±3.1 | 25.3±3.3 | 25.2±3.2 | 0.761 |
| TC (mmol/L) | 5.1±1.0 | 5.0±1.0 | 5.0±1.0 | 0.496 |
| TG (mmol/L) | 1.6±0.9 | 1.6±0.9 | 1.7±0.9 | 0.724 |
| HDL (mmol/L) | 1.4±0.3 | 1.4±0.3 | 1.4±0.3 | 0.949 |
| LDL (mmol/L) | 3.4±0.9 | 3.3±1.0 | 3.3±0.9 | 0.388 |
| APOE ε4 positive, n(%) | 44 (26.5) | 82(49.4) | 40(24.1) | 0.882 |
| Aβ_40_ (pg/mL) | 31.6±16.7 | 51.7±3.8 | 64.2±4.4 | <0.001 |
| Aβ_42_ (pg/mL) | 41±6.5 | 41.5±6.4 | 40±7.0 | 0.006 |
| MMSE at baseline | 25.8±3.7 | 26.2±3.6 | 26.0±3.4 | 0.257 |

*SBP, systolic blood pressure; BMI, body mass index; TC, total cholesterol; TG, triglycerides; HDL, high-density lipoprotein cholesterol; LDL, low-density lipoprotein cholesterol; APOE, apolipoprotein E; Aβ, Amyloid-β; MMSE, Mini-Mental State Examination.*
