# Supplementary material for: Non-linear Relationship Between Plasma Amyloid-β 40 Level and Cognitive Decline in a Cognitively Normal Population
Source: Front Aging Neurosci. 2020 Sep 11;12:557005. doi: 10.3389/fnagi.2020.557005 (PMC7516983; doi:10.3389/fnagi.2020.557005)
Supplement: Supplementary file 2 [file Table_2.docx]

**Table S2.** The relationship between plasma Aβ _40_ and cognitive decline.

| Models | No. of events (%) | OR | 95%CI | *p* |
| --- | --- | --- | --- | --- |
| Unadjusted |  |  |  |  |
| Categorical Aβ _40_^a^ |  |  |  |  |
| <47.2 pg/mL | 137 (33.4) | 0.75 | 0.57-1.00 | 0.053 |
| 47.2~55.6 pg/mL | 163 (40.0) | 1.0 (ref) | |  |
| ≥55.6 pg/mL | 156 (37.0) | 0.88 | 0.67-1.17 | 0.378 |
| Age-and sex-adjusted |  |  |  |  |
| Categorical Aβ _40_ |  |  |  |  |
| <47.2 pg/mL | 137 (33.4) | 0.75 | 0.56-1.00 | 0.051 |
| 47.2~55.6 pg/mL | 163 (40.0) | 1.0 (ref) | |  |
| ≥55.6 pg/mL | 156 (37.0) | 0.87 | 0.65-1.15 | 0.312 |
| Fully adjusted |  |  |  |  |
| Categorical Aβ _40_ |  |  |  |  |
| <47.2 pg/mL | 137 (33.4) | 0.73 | 0.55-0.98 | 0.034 |
| 47.2~55.6 pg/mL | 163 (40.0) | 1.0 (ref) | |  |
| ≥55.6 pg/mL | 156 (37.0) | 0.85 | 0.64-1.13 | 0.258 |

*OR, odds ratio; CI, confidence interval; Aβ, Amyloid-β.*

*^a^ Categorical Aβ _40_ was classified by 3 tertiles defined by the 33th percentiles*

*and 66th percentiles.*

**Article title**: Non-linear relationship between plasma Amyloid-β 40 level and cognitive decline in a cognitively normal population

**Journal name:** Journal of Neurology

**Author names:** Fan Gao, Suhang Shang, Chen Chen, Liangjun Dang, Ling Gao, Shan Wei, Jin Wang, Kang Huo, Meiying Deng, Jingyi Wang, Qiumin Qu

**Corresponding author:** Qiumin Qu, Department of Neurology, The First Affiliated Hospital of Xi’an Jiaotong University, 277 West Yanta Rd, Xi’an 710061, China. Tel./fax: +86 29 8532 4083. E-mail [quqiumin@126.com](mailto:quqiumin@126.com)
